# The body and the brain keep the score: a data-driven conceptual model linking trauma and postural tachycardia syndrome

**DOI:** 10.3389/fpsyg.2026.1829434

**Published:** 2026-06-09

**Authors:** Taylor B. Crouch, Gisela Chelimsky, Laura Boylan, Madison Maxwell, Grace Westcott, Spencer Owen Chase, Tammy Redman, James Burch, Raouf Gharbo, Mary Wells, Whitney Redemer, Patricia Kinser, Thomas Chelimsky

**Affiliations:** 1 Virginia Commonwealth University School of Medicine, Richmond, VA, United States; 2 Virginia Commonwealth University College of Humanities and Sciences, Richmond, VA, United States; 3 Virginia Commonwealth University School of Public Health, Richmond, VA, United States; 4 Virginia Commonwealth University School of Nursing, Richmond, VA, United States

**Keywords:** adverse childhood experiences, autonomic nervous system, neurobiology, postural tachycardia syndrome, trauma

## Abstract

Postural tachycardia syndrome (POTS) is a common, often disabling disorder of autonomic nervous system regulation without a unifying etiological account. Converging evidence suggests infectious, physical, and emotional threats frequently precede onset. We propose a hypothesis-generating model in which POTS may, in some individuals, involve threat-induced, centrally maintained disruption of brain–body communication that may be responsive to neuroplasticity-based behavioral medicine. In this paper, we synthesized multidisciplinary literature and our team’s data spanning (a) autonomic and central nervous system responses to threat and trauma, (b) neurological alterations associated with early life stress, (c) links between adverse childhood experiences and posttraumatic stress disorder with autonomic symptom burden, and (d) clinical developments targeting the threat system and autonomic regulation. These data suggest that chronic or overwhelming threat exposure is associated with sympathetic activation, reduced vagal tone, and neuroplastic alterations within cortico-limbic and brainstem networks that parallel POTS features (exaggerated tachycardia, autonomic rigidity, multisystem dysregulation). Preliminary data indicate individuals with higher trauma exposure and PTSD symptoms report greater autonomic symptom severity and poorer global health. Emerging imaging suggests a potentially important role for the periaqueductal gray (PAG), a midbrain hub for autonomic, cardiovascular, motor, and pain responses to threat, which may fail to reset after trauma, leaving the ANS in a sustained escape-mode (fight/flight/freeze) that increases POTS risk. Overall, these findings provide preliminary conceptual support for a unified hypothesis linking trauma, PAG-mediated threat responses, and sustained autonomic dysregulation in POTS, underscoring the importance of trauma-informed care. Behavioral interventions that target threat reduction and autonomic regulation such as rate variability biofeedback and neuroplasticity-oriented psychotherapies, may complement standard medical care. Prospective, longitudinal studies are needed to clarify causal pathways and identify responsive subgroups, and randomized clinical trials are required to establish the efficacy of nervous system-focused behavioral interventions.

## 1 Review of what we know—and do not know—about POTS

Postural tachycardia syndrome (POTS) is a disorder of the autonomic nervous system (ANS) that affects an estimated 1–3 million Americans, though the true prevalence is likely higher due to underdiagnosis (Shaw et al., 2019; Cline and Einhardt, 2022). POTS is characterized by chronic orthostatic intolerance (OI) and excessive heart rate (HR) increase (≥30 bpm in adults, ≥40 bpm in adolescents) upon standing (Fedorowski, 2019). Recent efforts to describe the lived experience of POTS have revealed significant functional impacts and health disparities, including diagnostic delays (Bourne et al., 2021b), symptom-related difficulties that impact economic and employment domains (Bourne et al., 2021a), and gender inequalities (Eftekhari et al., 2025). Individuals with POTS frequently experience invalidation of their symptoms (Knoop et al., 2024) and report a lower quality of life across multiple areas, such as physical, mental, and social well-being (Seeley et al., 2023). Women are disproportionately affected by POTS, though the reasons for this sex disparity are likely multifactorial and incompletely understood. Proposed contributors include sex hormone influences on autonomic and vascular regulation, higher prevalence of autoimmune disorders and connective tissue conditions such as hypermobile Ehlers-Danlos syndrome among women, as well as potential differences in immune response, neuroendocrine function, and sociocultural factors, including gender-based healthcare bias and symptom invalidation, may further contribute to delayed diagnosis and increased burden among women (Shaw et al., 2019; Bourne et al., 2021b). These factors underscore the importance of considering both biological and structural contributors when characterizing POTS epidemiology.

Given growing cases of POTS since the COVID-19 pandemic (Mallick et al., 2023; Dulal et al., 2025) and the complexities in its presentation, efforts are needed to better characterize mechanisms of the disorder beyond its definitional criteria. Historically, the heart rate increase from supine to standing seemed critical in the definition of POTS, but data shows that this heart rate increase does not seem to be fully pathognomic, as the clinical presentation of patients with POTS is similar to patients with overlapping comorbidities that experience lightheadedness without meeting criteria for POTS (Chelimsky et al., 2015). Research has shown that a substantial portion of healthy youth demonstrate orthostatic tachycardia that meets or exceeds the POTS criteria on a tilt-table test despite being asymptomatic (Singer et al., 2012), suggesting that abnormal heart rate changes can also reflect normal variation and does not fully explain the clinical presentation of individuals with POTS. Further, a majority of patients affected by POTS also report other symptoms beyond the diagnostic criteria, including chronic dizziness, fatigue, brain fog, nausea, and gastrointestinal problems (Sheldon et al., 2015; Fedorowski, 2019). POTS is frequently comorbid with conditions such as migraines, chronic fatigue syndrome, hypermobile Ehlers-Danlos Syndrome, fibromyalgia, and irritable bowel syndrome, which can complicate efforts to diagnose or achieve pathophysiological clarity due to overlapping symptoms (Boris, 2018; Shaw et al., 2019; Vernino et al., 2021). The complex, heterogenous presentation of POTS underscores the need for multidisciplinary investigation into its driving and maintaining factors to achieve a more impactful conceptualization and treatment framework.

POTS has historically been described and treated from a primarily biomedical framework, with most of the research on POTS from the last several decades focusing on peripheral abnormalities that characterize the disease presentation, such as cardiovascular, immunologic, and endocrine mechanisms (Fu et al., 2010; Watari et al., 2018). Despite important discoveries regarding potential structural and peripheral contributors to POTS, including hypovolemia, hyperadrenergic states, small fiber neuropathy, autoimmune and inflammatory processes, connective tissue abnormalities such as hypermobile Ehlers-Danlos syndrome, and cardiovascular deconditioning, it remains unclear whether these abnormalities represent primary causes, downstream consequences, or interacting contributors across patient subgroups (Benarroch, 2012; Vernino et al., 2021). Thus, recent research has begun to expand beyond the periphery to better understand the role of faulty central network connections as a potential driver of POTS (Umeda et al., 2015). Neuroimaging studies reveal alterations in brain regions responsible for autonomic regulation, emotional processing, and sensorimotor integration in individuals with POTS compared to healthy controls; moreover, certain differences, such as reduced insular volume, have been linked to trait anxiety and depression, indicating that these structural findings reflect meaningful variations in central regulatory systems (Umeda et al., 2015). The role of central conditioning has begun to receive increasing attention, with Norcliffe-Kauffmann and colleagues demonstrating that compared to healthy control subjects, patients with POTS had not only higher levels of somatic vigilance and anxiety, but also increased anticipatory tachycardia prior to upright positioning in a tilt-table test, an indicator of implicit fear conditioning (Norcliffe-Kaufmann et al., 2022). Given such findings and the frequent overlap with other chronic, centralized conditions, some have argued that POTS may be better characterized not as a structural cardiovascular condition, but as a disorder of the central nervous system (CNS) (Blitshteyn, 2022), explaining its linkage to chronic pain conditions (Chelimsky et al., 2015).

Existing biomedical models of POTS have identified multiple potential contributors, including hypovolemia, hyperadrenergic physiology, neuropathic mechanisms, autoimmune processes, connective tissue abnormalities, and cardiovascular deconditioning (Fu et al., 2010; Watari et al., 2018). However, the heterogeneity of these findings and absence of a unifying etiological account suggest that additional central mechanisms may also warrant consideration. Accordingly, this paper explores trauma- and threat-related alterations in central autonomic regulation as one potential complementary framework rather than a replacement for established biomedical models.

Despite many important discoveries about structural abnormalities, it is unknown whether they are causes or consequences of the disorder, and the etiology of POTS has not been established in even a subset of patients. Thus, recent research has begun to expand beyond the periphery to better understand the role of faulty central network connections as a potential driver of POTS (Umeda et al., 2015). Neuroimaging studies reveal alterations in brain regions responsible for autonomic regulation, emotional processing, and sensorimotor integration in individuals with POTS compared to healthy controls; moreover, certain differences, such as reduced insular volume, have been linked to trait anxiety and depression, indicating that these structural findings reflect meaningful variations in central regulatory systems (Umeda et al., 2015). The role of central conditioning has begun to receive increasing attention, with Norcliffe-Kauffmann and colleagues demonstrating that compared to healthy control subjects, patients with POTS had not only higher levels of somatic vigilance and anxiety, but also increased anticipatory tachycardia prior to upright positioning in a tilt-table test, an indicator of implicit fear conditioning (Norcliffe-Kaufmann et al., 2022). Given such findings and the frequent overlap with other chronic pain conditions and other disorders understood through centralized or neuroplastic frameworks (Chelimsky et al., 2015), some have argued that POTS may be better characterized not as a structural cardiovascular condition, but as a disorder of the CNS (Blitshteyn, 2022). These overlaps suggest—as will be explored in detail in the current paper—that for at least some individuals, POTS may share broader mechanisms involving central sensitization, maladaptive threat processing, altered interoception, and dysregulated brain–body communication rather than reflecting solely peripheral cardiovascular dysfunction. Of particular relevance, the periaqueductal gray (PAG), a midbrain structure central to autonomic defense, pain modulation, and threat processing, has been implicated both in chronic pain conditions and in emerging models of POTS pathophysiology. Given the PAG’s role in coordinating defensive autonomic responses, pain perception, and survival behaviors, dysfunction within these systems may represent a mechanistic bridge linking trauma, chronic pain, and autonomic dysregulation. Introducing this broader neurobiological context in this paper may help explain why frameworks developed in chronic pain and neuroplastic symptom conditions may hold translational relevance for understanding and treating certain POTS subgroups.

## 2 Conceptual model of the role of trauma and threat in POTS

POTS typically presents after a threat of some type, which may include a pathogenic infection such as COVID-19, a physical trauma such as a traumatic brain injury, or an emotional trauma (Heyer et al., 2016; Mallick et al., 2023). The type of preceding threat may produce a specific “phenotype” of POTS; for example, some have called for a distinct label of “long-COVID POTS” to describe POTS that develops in individuals with probable or confirmed history of SARS-CoV-2 infection in order to emphasize the impact of viral and immunologic pathways (Raj et al., 2021; Ståhlberg et al., 2021). However, there is no evidence that individuals who develop POTS after COVID-19 infection differ from those who develop POTS after other events, and there has been surprisingly little work to conceptually unify or classify the types of threat that typically precede the disorder. In this perspective paper, we present a conceptual framework that positions trauma as a potentially important contributor to POTS through the lens of an impact on CNS function, rather than the typical lens of an attack on the immune or cardiovascular systems. Importantly, greater conceptual precision is needed regarding the terms “threat” and “trauma,” which are related but distinct constructs within this framework. In the present manuscript, *threat* refers broadly to any internal or external stimulus, whether physical, infectious, psychological, or physiological—that is perceived by the nervous system as signaling potential danger and capable of activating defensive neurobiological responses (i.e., the “fight, flight, fawn, and freeze” system) (LeDoux, 1996, 2012). Threats may be acute or chronic, objective or perceived, and need not result in lasting dysregulation. In contrast, *trauma* refers more specifically to overwhelming, chronic, or inadequately resolved threat exposure that produces persistent maladaptive alterations in autonomic, neuroendocrine, emotional, or neurobiological regulation. Thus, while all trauma involves threat, not all threats become traumatic. This distinction is important because POTS may be precipitated by a range of threats (e.g., viral illness, injury, psychological stress), whereas trauma may represent one pathway through which certain threats exert longer-term predisposing or maintaining effects on nervous system regulation. Clarifying this distinction enhances precision, supports testability, and better reflects the heterogeneity of pathways potentially contributing to POTS.

Importantly, trauma may influence POTS development through several distinct but potentially interacting roles, which warrant conceptual separation for clarity and future empirical testing. First, trauma may function as a *predisposing factor*, wherein early life adversity, chronic stress exposure, or prior trauma contributes to long-term alterations in autonomic regulation, threat detection systems, and neurobiological vulnerability, thereby increasing susceptibility to later dysautonomia. Second, trauma may serve as a *precipitating factor*, in which an acute threat, whether infectious, physical, or psychological, triggers onset of POTS symptoms in biologically or psychologically susceptible individuals. Third, trauma may operate as a *maintaining or exacerbating factor*, wherein ongoing hypervigilance, maladaptive threat prediction, chronic stress, healthcare-related invalidation, or unresolved trauma responses contribute to symptom persistence, autonomic rigidity, or relapse over time. These pathways are not mutually exclusive and may co-occur or vary substantially across individuals, potentially contributing to the heterogeneity observed in POTS presentations. This framework emphasizes that trauma is neither necessary nor sufficient for POTS development, but rather may represent one of multiple interacting contributors within a broader biopsychosocial and neurophysiological model.

## 3 Pathophysiology—impact of threat on the ANS and CNS

Exploring the role of trauma in POTS requires a basic understanding of how the human body responds physiologically to threats. Interconnected systems in the CNS and autonomic nervous system (ANS) allow humans to subconsciously monitor the internal and external environment for indicators of danger (Porges, 2003, 2004), and detection of threat occurs through fast, subcortical neurobiological pathways that allow for sensory input to trigger activation of the amygdala, brainstem, and hypothalamus. In other words, this “low-road” appraisal system allows the brain to initiate a defensive response before conscious awareness of a threat (LeDoux, 1996, 2000). While this allows for evolutionary survival, it also prioritizes speed over precision, meaning that neutral or non-threatening stimuli can be misinterpreted as dangerous, triggering defensive pathways even in the absence of actual harm (Pessoa and Adolphs, 2010). Once the threat is detected, the ANS rapidly shifts into a survival mode. Sympathetic activation increases heart rate, blood pressure, and respiration to prepare humans for action, while the parasympathetic nervous system (PNS) withdraws (Thayer and Lane, 2000; Porges, 2007). In overwhelming or inescapable threat conditions, survival responses may move toward immobilization, characterized by bradycardia, metabolic down-regulation, and behavioral shutdown (Porges, 2007, 2009). These shifts in the ANS are adaptive during acute threat, but when repeatedly activated, they have been shown to lead to persistent sympathetic activation (PSA) and autonomic rigidity, which are linked to increased allostatic load and morbidity (McEwen, 2017).

ANS stress pathways are also tightly linked to CNS processes. The hypothalamic–pituitary–adrenal (HPA) axis releases glucocorticoids that allow individuals to sustain vigilance and energy mobilization to body organs and muscles associated with “fight-or-flight” activity (Herman et al., 2016). As the brain’s main goal is survival, neural pathways that are repeatedly used begin to “wire together” to allow for faster activation (Hebb, 2002). In the context of chronic threat activation, humans become increasingly good at detecting and responding to threats (i.e., hypervigilance). As these neural circuits develop increasing bias toward rapid threat detection and to promote survival, the neural circuits involved in inhibitory control and self-regulation begin to weaken (Arnsten, 2009; McEwen and Morrison, 2013). This consolidation of threat-responsive pathways biases our ANS and CNS toward hypervigilance and rapid reactivity, which becomes maladaptive in the absence of objective danger (Milad and Quirk, 2012; McEwen and Morrison, 2013).

Thus, chronic stress and trauma produce structural and functional alterations in cortico-limbic regions that are maladaptive for autonomic and emotion regulation including amygdala hyperreactivity, reduced medial prefrontal cortex and anterior cingulate cortex regulation, hippocampal volume reductions, altered insular processing, and changes within the brainstem’s autonomic centers (Teicher and Samson, 2016; Ilomäki et al., 2022). Moreover, chronic stress and trauma have also been shown to weaken integration of fronto-limibc, salience, and default mode networks, as well as disrupt coherence within the central autonomic network (Herringa, 2017; Olson et al., 2019). Collectively, these findings illustrate that repeated threat may contribute to reorganization of both ANS and CNS systems toward persistent defensive activation for the benefits toward acute survival which give way to long-term losses in regulatory flexibility, physiological recovery, and often lead to individuals maintaining a state of PSA. Such maladaptive central-autonomic coupling provides a mechanistic foundation for understanding how threats can precipitate or exacerbate disorders such as POTS, which epitomizes the hypersensitive sympathetic response pattern (i.e., exaggerated heart rate responses to standing, exercise and other stimuli, excessive adrenergic responses, and sometimes exaggerated diastolic pressure rise with standing) and provides a true window into this pathophysiologic cascade.

## 4 Spotlight on the PAG: established functions and hypothesized relevance to POTS

The Periaqueductal Gray (PAG) is a small brain structure in the brain stem that plays a critical role in threat response. Generally speaking, the dorsal portion of the PAG is responsible for activating the fight-or-flight response if a threat is implicitly perceived as survivable, and the ventral portion is responsible for the freeze response if the threat is perceived as unsurvivable. The “fight” response requires an increase in blood pressure and heart rate, vasoconstriction of the legs and the renal system, and extracranial vasodilation to mobilize for “attack.” In a “flight” response, the body also responds with an increase in blood pressure and heart rate, but the legs also need vasodilation with vasoconstriction of the extracranial areas and the kidneys. If the brain predicts that the person will not survive a threat, the PAG prepares the subject for death, by providing opioid mediated analgesia, hypotension, bradycardia and paralysis (Bandler et al., 1991a; Bandler et al., 1991b). Beyond responding to threats, the PAG is also implicated in social interactions, pro-sexual behavior, the intake of food and water, and control of speech (Silva and McNaughton, 2019).

The PAG may play a key role in the development of POTS and comorbid chronic conditions. Under this hypothesized mechanism, if a threat is perceived as survivable, the dorsal (fight-or-flight) portion of the PAG is triggered, directly producing a sympathetic response that can become persistent. If a threat is perceived as unsurvivable, the PAG will prepare the person for death, which can present as paralysis (“freezing”) with hypotension that can then trigger a compensatory sympathetic response over time. To further explore this hypothesis, we are using functional MRI (fMRI) to compare cortical brain regions activated in adolescents with POTS versus healthy controls during a looming task, a research paradigm that involves an approaching predator as a visual stimulus to activate subconscious threat responses. Our preliminary data shows that the PAG is activated as expected in healthy controls and POTS subjects alike during the looming task, but the linked cortical brain regions activated differ between the two groups (Tavinogradov et al., 2025). For POTS patients, the task activated the right amygdala and the right insula, whereas the right superior anterior cingulate cortex and left insula were activated in healthy controls. These findings support the idea that incoming threats are channeled through decision-making circuits in healthy control subjects, whereas they are channeled into right sided emotional-limbic circuits in POTS subjects, which may impede self-regulatory capacity.

Importantly, while the PAG’s general neurobiological role is well established, its specific contribution to POTS pathophysiology remains preliminary and largely hypothesis-generating. Our proposed model suggests that maladaptive PAG-mediated threat responses or impaired recalibration following significant threat exposure may represent one potential mechanism contributing to persistent autonomic dysregulation in certain individuals, but this hypothesis requires further empirical validation. Emerging neuroimaging findings, including our preliminary looming-task data, suggest altered PAG-linked circuitry in POTS populations; however, these findings should be interpreted cautiously until replicated in larger, prospective studies. Thus, PAG dysfunction should currently be understood not as a confirmed causal mechanism of POTS, but rather as a promising and biologically plausible framework for future investigation that may help integrate trauma, autonomic dysregulation, chronic pain overlap, and central nervous system contributions in some patient subgroups.

## 5 Risk factors for persistent pathophysiological response to threat

Clearly, many individuals experience traumas and threats with activation of these survival responses but without getting “stuck” in such a response, while others may go on to develop POTS or another chronic condition marked by chronic autonomic dysregulation. While the risk factors for getting “stuck” in a threat response have been understudied in POTS populations specifically, there is a large body of literature indicating that some individuals are broadly at higher risk for persistent nervous system dysregulation. For example, certain personality characteristics like neuroticism (Čukić and Bates, 2015) and perfectionism (Azam et al., 2015), as well as neurodivergence (Thapa et al., 2019; Robe et al., 2021; Villar de Araujo et al., 2023), have been linked to indicators of PSA including lower heart rate variability (HRV) and altered autonomic tone. Additionally, there is extensive evidence that childhood trauma and adversity are linked to lasting autonomic dysregulation, which will be detailed in the following section. It is likely that a threat or trauma is more likely to trigger a persistent pattern of nervous system dysregulation for individuals who have these or other risk factors, though further research is needed to clarify these links in POTS patients specifically.

## 6 Links between trauma and nervous system dysregulation and autonomic symptom burden

There is extensive literature linking trauma—including early life trauma and PTSD—with long-term changes in autonomic, neuroendocrine, and neuroimmune systems (Schneider and Schwerdtfeger, 2020; Smith and Pollak, 2020; Siciliano et al., 2022; Wesarg et al., 2022). While most of this research on trauma-related autonomic dysregulation has been conducted outside of POTS, it illuminates biologically plausible pathways that could link trauma and development of the disorder. Adverse childhood experiences (ACEs)—which include instances of abuse, neglect, or household dysfunction prior to age 18—are of critical importance given that the threat presents during periods of rapid neurobiological maturation (Smith and Pollak, 2020). While the immature brain adapts to stress, these adaptations may have consequences over the long-term. Individuals exposed to chronic threat and stress during this time have been shown to have lower resting HRV, reduced vagal tone, and greater sympathetic reactivity—all of which biases the system toward detecting and responding to threat (Rudd et al., 2021; Campbell, 2022; Wesarg et al., 2022). Recent research has shown that autonomic dysregulation mediates the association between childhood trauma and chronic pain vulnerability (Costa et al., 2025), highlighting its mechanistic role between early stress and physiological consequences. ACEs have also been associated with altered cortisol rhythms, blunted, or dysregulated stress responses, abnormal frontolimbic circuitry, and elevated baseline inflammation (Herringa, 2017; Ouellet-Morin et al., 2019; Rudd et al., 2021). Further, longitudinal studies have demonstrated that the physiological impacts of ACEs can persist into adulthood and increase risk for stress-related physical and psychological pathology (Rudd et al., 2021). While ACEs have received the most research attention, it is also important to note that early life stress that may not cross the threshold of abuse or neglect, such as chronic relational traumas (e.g., inconsistent or immature emotional responsiveness from a caregiver; chronic feelings of being misunderstood or judged), have also been linked to long-term ANS dysregulation and reduced regulatory flexibility (Maunder and Hunter, 2001; Luyten and Fonagy, 2018).

These nervous system changes and alterations in brain–body regulation systems have also been observed in adults after exposure to trauma, particularly among individuals who develop PTSD (Schneider and Schwerdtfeger, 2020). Autonomic profiles of individuals with PTSD closely mirror those seen in individuals with significant childhood adversities (e.g., low resting HRV, reduced vagal tone, and exaggerated sympathetic reactivity) (Ge et al., 2020; Schneider and Schwerdtfeger, 2020). PTSD is also associated with HPA-axis dysregulation and chronic inflammatory activation, which also parallels findings from the ACEs literature (Schumacher et al., 2019). These patterns suggest that both early- and adult-onset trauma can produce maladaptive effects on the body’s stress-regulation systems. Further, the experience itself of having chronic illness can be traumatic—whether from the diagnostic process, hospitalizations, invasive medical procedures, loss of autonomy, or experiences of discrimination or invalidation from healthcare providers (Pacella et al., 2013; Cyr et al., 2021)—thereby potentially serving as another triggering and/or compounding stressor on the sensitized nervous system.

Collectively, the literature on ACEs and PTSD highlights the long-term, multisystem impact of chronic, overwhelming threat exposure on the neural and physiological functioning. These alterations increase risk not only for mental-health disorders but also for a range of physical symptoms (e.g., headaches, musculoskeletal pain, gastrointestinal disturbances, fatigue, and autonomic discomfort) that are frequently reported among individuals with POTS (Benarroch, 2012). For example, a meta-analysis conducted in 2017 found significantly higher rates of chronic pain among individuals with PTSD compared to those without trauma exposure (Siqveland et al., 2017), underscoring the link between trauma-related neurobiology and somatic symptom expression. These parallels strengthen the biological plausibility that trauma may act as one of many sensitizing or underlying factors in the development or maintenance of POTS.

We conducted preliminary investigation into the impact of ACEs and PTSD on autonomic symptom burden in individuals who do develop autonomic disorders like POTS and have found notable links between childhood trauma, PTSD, and higher autonomic symptomatology (Crouch et al., 2024). Specifically, we examined baseline levels of self-reported POTS symptom severity (as measured by the Malmö POTS symptom score, which has a clinical significance cutoff value of ≥42 to identify likely POTS) (Spahic et al., 2023) and general quality of life (the PROMIS Global Health Scale) along with self-reported measures of childhood adversity (Adverse Childhood Experiences questionnaires) and posttraumatic stress disorder (the PTSD subscale of the Millon® Clinical Multiaxial Inventory-IV, which is based on DSM-5 criteria) in 98 adults with dysautonomia (POTS not necessarily confirmed) entering our Comprehensive Autonomic Clinic (CAC). The mean ACEs score of the sample was 2.44 (SD = 2.732), and 30.5% met the clinical significance cutoff score of 4 or more. Both of these figures represent ACE exposures higher than those observed in general population samples (Swedo et al., 2023), while also highlighting that ACEs are far from a necessary or sufficient explanation for the development of POTS. A quarter of patients (25.5%) were significant for PTSD on the MCMI-IV, which is notably higher than the 3.6% national average for PTSD prevalence in adults (National Institute of Mental Health, 2019). ACEs and Malmö scores were significantly and positively correlated (*p* = 0.041, *R* = 0.207), and participants positive for PTSD reported significantly higher Malmö scores than those who did not (*p* < 0.001, *t* = −3.663), with means of 87.16 vs. 64.41, respectively. Further, individuals positive for PTSD were more likely to fall in the “poor” range of the PROMIS Global Health Scale, while individuals negative for PTSD were more likely to fall in the “fair” range.

These associations certainly do not prove a cause-effect relationship between trauma and POTS, nor are we proponents of such a simplistic explanation. However, the associations support a model in which continued activation of survival circuits bias the brain toward maladaptive persistent sympathetic activation with failure to restore healthy parasympathetic (vagal) dominance. While we are conducting active research on understanding these mechanisms, which are not currently confirmed, our findings, taken with the collection of literature across several disciplines suggests several possibilities: (1) Chronic autonomic imbalance after trauma biases toward sympathetic dominance and reduced vagal tone, which predisposes to exaggerated and sustained autonomic reactivity in response to a threat (e.g., a virus, injury, or emotional trauma), which may increase vulnerability to POTS under the “right” circumstances; (2) Trauma exposure may alter limbic and brainstem circuits that control autonomic output (insula, amygdala, prefrontal cortex), potentially changing central “set-points” for cardiovascular control; (3) Trauma dysregulates the HPA axis and immune signaling such that inflammatory mediators and altered neuroendocrine responses affect vascular tone and baroreflex sensitivity; and (4) Trauma may act as a precipitating/stressing event in individuals with predisposing factors (e.g., genetic, small-fiber neuropathy, volume regulation abnormalities, etc.), producing a POTS phenotype in some but not all. These differing pathways to POTS are not presumed to be mutually exclusive, and in fact may differ in different individuals, with one pathway dominating in one individual compared to another.

These varying pathways may also help explain the substantial heterogeneity observed within POTS populations and support conceptualization of POTS not as a singular disorder, but potentially as a syndrome with overlapping yet mechanistically distinct subtypes. For example, some individuals may present with predominantly *post-viral or immune-mediated subtypes*, in which infection-triggered inflammatory or autoimmune mechanisms play a more central role; others may exhibit *neuropathic subtypes*, characterized by peripheral autonomic denervation or small fiber dysfunction; still others may reflect *hyperadrenergic or centrally mediated subtypes*, in which maladaptive threat processing, trauma-related autonomic sensitization, or altered central autonomic network functioning may play a larger role. Physical injury, traumatic brain injury, connective tissue disorders, and endocrine abnormalities may also represent distinct or interacting mechanistic contributors. These subtype distinctions are unlikely to be mutually exclusive, and many patients may exhibit mixed presentations. However, recognizing heterogeneity may improve both scientific precision and treatment matching, as individuals with greater evidence of central sensitization, trauma-related dysregulation, or conditioned autonomic responses may benefit more from nervous system-focused behavioral interventions such as HRV biofeedback, PORT, or somatic therapies, whereas other subgroups may require more targeted biomedical or immunologic approaches. Future research aimed at refining biologically and clinically meaningful POTS subtypes may enhance personalized intervention development and improve outcomes across this heterogeneous population.

These hypothesized mechanisms may also be further contextualized through predictive coding and Free Energy Principle frameworks, which offer complementary models for understanding how maladaptive threat-related priors may contribute to persistent autonomic dysregulation (Friston, 2010; Owens et al., 2018). Within this framework, the brain continuously generates predictions about internal bodily states and updates these predictions through incoming interoceptive signals in an effort to minimize prediction error and maintain physiological homeostasis. Trauma exposure may bias these predictive systems toward heightened threat expectancy, such that prior beliefs emphasizing danger or physiological vulnerability become overly weighted relative to corrective sensory input. In this model, trauma-associated alterations in limbic, insular, prefrontal, and brainstem circuits may contribute not only to sympathetic dominance and reduced vagal tone, but also to impaired hierarchical error correction, wherein threat-related autonomic predictions persist despite the absence of ongoing danger. This sustained overestimation of threat may reinforce hypervigilance, maladaptive autonomic set-points, and exaggerated cardiovascular responses to otherwise benign physiological stimuli, such as orthostatic challenge. Thus, POTS in some individuals may reflect, in part, a disorder of disrupted interoceptive inference in which trauma-conditioned defensive priors perpetuate autonomic rigidity and false alarm responses. Integrating predictive coding perspectives strengthens the theoretical foundation for understanding POTS as a centrally mediated disorder of brain–body regulation while further supporting interventions aimed at recalibrating maladaptive threat predictions and restoring autonomic flexibility.

Distinguishing trauma’s role as predisposing, precipitating, or maintaining may also improve testability of this conceptual model. For example, prospective studies could examine whether early adversity predicts increased autonomic vulnerability, whether discrete trauma exposures more commonly precede symptom onset, or whether unresolved trauma symptomatology predicts poorer symptom persistence or treatment responsiveness. Such distinctions may enhance precision in both mechanistic research and intervention development.

## 7 The role of behavioral approaches in addressing autonomic nervous system dysregulation

Broadly speaking, the lack of a unifying understanding of POTS pathophysiology has contributed to the limited effectiveness of standard treatment strategies (Vernino et al., 2021), which focus primarily on symptom management and targeting individual peripheral abnormalities, e.g., with medications, salt and fluid supplementation, and compression garments (Sheldon et al., 2015). As detailed in prior sections, threat and trauma can lead to persistent sympathetic activation in the ANS such as that seen in POTS, but standard POTS treatments do not address the threat system, despite experimental evidence of the role of fear conditioning in POTS (Norcliffe-Kaufmann et al., 2022). Further, there is evidence that POTS is commonly characterized not only by inappropriate sympathetic activity but also a variety of other symptoms consistent with CNS dysregulation, such as fatigue, brain fog, anxiety, GI distress, and others that are not targeted by standard POTS treatments. From the lens of the current paper, treatments should aim to address not only individual symptom management (e.g., beta blockers to lower heart rate), but also the underlying system of autonomic dysregulation that lives in the mind/body interface to lead to more lasting change. Fortunately, while the investigation into mind/body behavioral treatments for POTS is still nascent, there is some promising evidence that supports ongoing testing of such treatments. First, there is accumulating evidence of autonomic imbalance marked by lower HRV in patients with POTS (Swai et al., 2019), and comorbid disorders like long-COVID (Suh et al., 2023), and more importantly, that HRV and related autonomic measures are predictors of improved responsiveness to POTS treatment (Wang et al., 2019; Cui et al., 2022; Yuan et al., 2023). For example, HRV biofeedback (HRVB) combines a paced breathing regimen (~5–6 breathes per minute) with monitoring of heart rate and respiration to establish a quantifiable, rhythmic pattern known as “HRV coherence.” Coaching and biofeedback are used to ensure that patients learn to synchronize their breathing and heart rate, and when this occurs, patients typically self-report meaningful experiential shifts (e.g., feelings of calmness or gratitude) (Lehrer and Gevirtz, 2014). The clinical utility of HRVB for treating POTS patients specifically has been recognized in multiple studies (Schnekenberg et al., 2023; Corrado et al., 2024; Mauro et al., 2024). Further, prior research has demonstrated that HRVB reduces comorbid symptoms commonly observed among POTS patients, including stress and pain (Berry et al., 2014), anxiety, depression, insomnia, and post-traumatic stress (Nagpal et al., 2013). HRVB also improves emotional regulation (i.e., resilience) and cognitive function (Bradley et al., 2010; Park and Thayer, 2014). These observations suggest that interventions designed to restore autonomic homeostasis and increase HRV may alleviate POTS symptoms, which lends further support to autonomic imbalance being centrally important in POTS.

In addition to “bottom-up” strategies like HRVB, our team has been developing and testing more “top-down” psychotherapeutic strategies that aim to reduce the brain’s perception of threat, thereby promoting greater regulation of the nervous system. This approach, which we have termed POTS Reprocessing Therapy (PORT), was adapted from Pain Reprocessing Therapy (PRT), an evidence-based treatment for centralized pain conditions, given our hypothesis that POTS represents a different manifestation of a similar functional dysregulation in the nervous system (Crouch et al., 2026). In centralized pain conditions such as fibromyalgia, pain is understood to be an implicitly learned “false alarm” signal that occurs in the absence of ongoing peripheral tissue damage to the body (Clauw, 2014). We propose that the heart rate elevation and dizziness experienced by POTS patients may represent a similar “false alarm,” wherein the brain and heart are structurally healthy, but the CNS activates an autonomic threat response in response to a benign trigger (standing). The rationale for PORT is informed not solely by analogy to chronic pain treatment, but by converging mechanistic parallels between POTS and other centralized or neuroplastic symptom conditions. Centralized pain models propose that persistent symptoms may, in some individuals, reflect maladaptive threat appraisal, heightened interoceptive sensitivity, conditioned physiological responses, and dysregulated central nervous system signaling rather than ongoing peripheral tissue damage alone (Clauw, 2014; Ashar et al., 2022). Similar mechanisms may plausibly contribute to POTS symptom persistence in relevant subgroups, particularly given evidence of heightened somatic vigilance, anticipatory autonomic responses, altered central autonomic network functioning, and frequent overlap with other centralized conditions such as fibromyalgia, chronic fatigue syndrome, and functional neurological disorders. In this framework, orthostatic symptoms may, in some cases, become reinforced through maladaptive predictive coding, fear conditioning, and sustained autonomic threat responses, even when the initial precipitating trigger was biomedical in nature. Thus, PORT seeks to target hypothesized top-down contributors including maladaptive threat appraisal, conditioned autonomic reactivity, and false alarm signaling while complementing, rather than replacing, standard biomedical management. By integrating principles of safety learning, symptom reinterpretation, interoceptive recalibration, and autonomic flexibility, PORT is designed as a theoretically informed adjunctive intervention aimed at reducing centrally mediated amplification of symptoms for appropriately selected patients. This framework does not suggest that all POTS is centrally driven, but rather proposes a potential neuroplastic pathway relevant for certain individuals within this heterogeneous population.

Given PRT’s effectiveness in reducing pain, it is reasonable to apply similar principles to patients with POTS, with the expectation of comparable therapeutic outcomes. The PORT treatment program is designed to educate patients about the nervous system’s role in the development and maintenance of symptoms. It emphasizes how trauma, whether caused by a pathogen, physiological injury, or emotionally charged event, can trigger a shift into PSA. Building on this foundational understanding, PORT teaches patients skills to reduce threat signals to the brain and activate the ventral vagal pathway. This activation supports a return to social engagement, connectedness, coregulation, and physiological restoration. By addressing these underlying neurobiological mechanisms, PORT aims to help regulate nervous system responses and alleviate symptoms. Unlike cognitive behavioral therapy (CBT), which focuses on reframing thoughts and learning coping strategies, PORT is designed to target hypothesized mechanisms and therefore symptom regulation more directly. Still aligned with evidence-based practices, PORT incorporates a cognitive component that encourages safety reappraisal of symptoms. Patients learn to reinterpret physiological sensations while engaging in parasympathetically activating interventions, further supporting nervous system regulation. In essence, PORT integrates both top-down (cognitive) and bottom-up (physiological) processing with the aim of mitigating conditioned threat responses in the nervous system.

In addition to HRV biofeedback and neuroplasticity-oriented psychotherapies such as PORT, other body-focused trauma therapies may also hold relevance for subsets of patients with POTS, particularly those with significant trauma histories or pronounced autonomic dysregulation. Somatic therapies such as Somatic Experiencing emphasize interoceptive awareness, autonomic nervous system regulation, and gradual processing of unresolved defensive responses through bottom-up physiological mechanisms rather than primarily cognitive restructuring (Payne et al., 2015). These approaches aim to restore autonomic flexibility by supporting completion of maladaptive fight, flight, or freeze responses and recalibrating nervous system responses to internal and external cues. Preliminary evidence suggests potential benefits of such interventions in trauma-related disorders, chronic pain, and conditions characterized by dysregulated autonomic and stress-response systems (Andersen et al., 2017). Although empirical investigation of somatic trauma therapies in POTS remains limited, their mechanistic overlap with the current conceptual model, including emphasis on interoception, autonomic recalibration, and restoration of regulatory flexibility, suggests they may represent promising complementary avenues for future clinical research. Further investigation is warranted to determine whether such body-focused trauma interventions may enhance outcomes for individuals whose POTS symptomatology is meaningfully shaped by trauma-related nervous system sensitization.

While further testing is still needed to assess the efficacy of PORT and other mind/body approaches for treating POTS, the established evidence supporting HRVB provides foundational support for the argument that restoring vagal activity via self-regulation skills reduces POTS symptoms. Further, there is evidence from the chronic pain literature that similar approaches such as Pain Reprocessing Therapy (Ashar et al., 2022) and Emotional Awareness and Expression Therapy (Yarns et al., 2024) improve physical symptoms through addressing the underlying associated fear circuits or emotional dysregulation and suppression circuits, respectively. Collectively, these advancements offer a promising invitation to expand our thinking around how a medical disorder like POTS could be treated more effectively.

## 8 Implications and trauma-informed treatment considerations

The present findings highlight not only the potential utility of POTS-specific behavioral medicine treatments, but also more broadly the need for trauma-informed approaches across both the psychological and medical treatment of POTS patients. Importantly, trauma-informed care does not imply that trauma-focused intervention is indicated or necessary for all patients, but rather that healthcare practitioners should strive to build a foundation of understanding and safety to support healing given what we know about the prevalence and impacts of trauma. Raja et al.’s (2015) trauma-informed care pyramid, combined with the evidence summarized above linking threat and trauma with POTS and other autonomic disorders, provides the basis for these recommendations. First, patient-centered communication should be a foundational element of trauma-informed care in this population, particularly given the high rates of invalidation in healthcare experiences reported among patients with autonomic symptoms (Wang et al., 2021). It is important that clinicians understand and communicate that patients’ symptoms are very real, regardless of whether and how trauma may have played a role in their onset and/or maintenance. Clinicians can promote safety and collaboration by using empathic and validating language (e.g., “These symptoms are real and may relate to how your body is processing stress”), making continual requests for consent during examinations, and providing sufficient time for patient questions. Moreover, communication emphasizing patient autonomy (e.g., “Let me know if this resonates with your experience”) may help counteract powerlessness often felt by patients with trauma (Raja et al., 2015; Armstrong et al., 2021).

Trauma-informed care for POTS patients also requires clinicians to understand and explain how trauma and emotion dysregulation can shape physiology, health behavior, and symptom trajectories. Psychoeducation that outlines the mind–body connection can help patients—and providers, if needed—reframe symptoms not as “just anxiety” or “all in their head,” but rather as manifestations of a sensitized but adaptable and retrainable nervous system. Behavioral recommendations (e.g., exercise, hydration, symptom tracking) can be presented as forms of nervous system retraining that promote self-regulation, body awareness, safety learning, and self-compassion. Additionally, clinicians should recognize that symptom flares may originate or become heightened during periods of stress and that behaviors sometimes labeled as “avoidance” or “noncompliance” may reflect learned emotional protective strategies rather than “resistance” to treatment (Raja et al., 2015). This shift in interpretation reduces stigma, supports more compassionate and collaborative treatment, and is aligned with a goal of enhancing nervous system safety.

Trauma-informed care also depends on coordinated work across interdisciplinary teams serving POTS patients. It is believed that mutual learning across disciplines (e.g., psychologists sharing trauma frameworks and PRT/PORT approaches, physicians sharing physiological insights, etc.) will optimize treatment efficacy and coherence. Treatment coherence is also supported through providers’ use of consistent language around autonomic regulation, as this helps prevent potential re-traumatization via confusing or invalidating encounters within the healthcare system (Center for Substance Abuse Treatment (US), 2014). Interdisciplinary case formulation should integrate medical data, autonomic markers, and psychosocial context, and regular team communication can ensure that appropriate referrals are made when trauma or emotional factors warrant additional psychological intervention (Raja et al., 2015).

Finally, trauma-informed practice includes awareness of clinicians’ own reactions and thoughtful use of trauma screening. Many physicians cite personal stress or personal trauma histories as barriers to trauma-informed implementation, highlighting the importance of monitoring countertransference and engaging in reflection or supervision to mitigate burnout and vicarious traumatization (Raja et al., 2015; Novilla et al., 2024). Screening for trauma using brief, sensitive tools (e.g., ACEs, PC-PTSD-5) can help contextualize autonomic symptoms, but must be paired with careful evaluation for structural causes such as cardiologic, genetic, endocrine, or metabolic conditions, to avoid misattribution (Dahm et al., 2023). Together, these practices position clinicians to deliver comprehensive, validating, and physiologically-informed care to patients with POTS and other autonomic disorders.

## 9 Methodological considerations and research gaps

Several limitations of the existing literature and of the preliminary data summarized in this paper warrant careful consideration. First, much of the evidence linking trauma, threat-related processes, and autonomic dysregulation in POTS is indirect, drawn from adjacent fields such as chronic pain, PTSD, and affective neuroscience. Although these bodies of work provide strong biological plausibility, they do not yet establish trauma as a causal mechanism in POTS. Accordingly, conclusions should be interpreted as hypothesis-generating rather than definitive. A major limitation of the current evidence base is the scarcity of prospective cohort studies that follow trauma-exposed individuals over time to determine who subsequently develops POTS. Most available data are retrospective or cross-sectional, limiting causal inference and raising the possibility of recall bias, reverse causation, or shared vulnerability factors. While associations between trauma exposure, PTSD symptoms, and greater autonomic symptom burden have been observed, these findings cannot determine whether trauma precedes autonomic dysregulation, exacerbates existing vulnerability, or reflects downstream consequences of chronic illness and healthcare-related stress. Confounding by comorbid medical conditions also represents an important limitation. Disorders commonly comorbid with POTS, such as autoimmune disease, connective tissue disorders, migraine, chronic fatigue syndrome, and long COVID, may independently influence both trauma exposure and autonomic nervous system function. These overlapping conditions complicate efforts to isolate trauma-specific mechanisms and underscore the likelihood that multiple, interacting pathways contribute to the heterogeneous presentation of POTS. Taken together, these limitations highlight the need for longitudinal, multimodal research integrating prospective trauma assessment, objective autonomic biomarkers, neuroimaging, and careful phenotyping of comorbid conditions. Such approaches may clarify whether trauma functions as a precipitating event, a maintaining factor, or a sensitizing influence within a broader predisposition-plus-trigger model. Additionally, research should aim to understand not only risk factors like ACEs but also the role of protective factors such as positive social roles and support in childhood to better characterize markers of resilience (Pugh et al., 2023), given that many individuals experience significant trauma but go on to thrive, physiologically and emotionally (Galatzer-Levy et al., 2018).

The preliminary clinical data described herein should also be interpreted in light of limitations, including reliance on self-report measures, absence of diagnostic confirmation for all cases, and limited demographic diversity (largely Caucasian women). Although the sample characteristics reflect typical POTS clinic populations, generalizability to more diverse populations remains uncertain. Structural and sociocultural stressors (e.g., discrimination, medical racism) represent understudied threat exposures that are not reflected in ACEs and are more likely to impact minoritized individuals. Additionally, the PORT treatment described above is still early in development and requires further testing via randomized controlled trials—which are currently underway—to establish whether it may effectively treat POTS symptoms.

## 10 Toward a trauma-informed conceptual model of POTS

The evidence outlined in this paper, taken together, suggest a hypothesis-generating conceptual framework linking trauma and POTS. Via the PAG, trauma may set the central autonomic network into one of its escape modes, such as fight, flight or freeze; failure to fully recalibrate the autonomic network back to a healthy operating mode after the trauma has passed may contribute to a state of persistent sympathetic activation for predisposed individuals, in turn leading to a dysregulated brain–body communication system that leads to manifestations of ANS dysregulation that increase the risk of a POTS diagnosis. Fortunately, preliminary evidence suggests POTS may respond to interventions that specifically address these pathophysiological processes. While more research is needed to clarify these complex mechanisms and confirm efficacy of the noted mind/body interventions for POTS, the findings detailed in this study already support the critical importance of trauma-informed care in this population. It is vital to note that the idea that POTS may have a brain origin or be linked to trauma does not “demote” this disorder to an “imagined” or non-real category or indicate that POTS is a psychological disorder, any more than any other disorder with a brain basis such as Parkinson’s disease, stroke, or multiple sclerosis. On the contrary, finding the true basis of very real POTS symptoms could help inform more targeted therapeutic developments for the thousands upon thousands of individuals suffering with this disorder.
